# Supplementary material for: A Low-Cost Electrochemical Method for the Determination of Sulfadiazine in Aquaculture Wastewater
Source: Int J Environ Res Public Health. 2022 Dec 16;19(24):16945. doi: 10.3390/ijerph192416945 (PMC9779263; doi:10.3390/ijerph192416945)
Supplement: Supplementary file 1 [file ijerph-19-16945-s001.zip › ijerph-2058366-supplementary.pdf]

Supplementary Information

A low-cost electrochemical method for the determination of sulfadiazine in aquaculture wastewater

Yang Liu [1,2], Jianlei Chen [2], Haiyan Hu [3], Keming Qu [2], Zhengguo Cui [2,*]

1 Faculty of Fisheries, Zhejiang Ocean University, Zhoushan 316022, China

2 Key Laboratory of Sustainable Development of Marine Fisheries, Ministry of Agriculture and Rural Affairs, Yellow Sea Fisheries Research Institute, Chinese Academy of Fishery Sciences, Qingdao 266071, China

3 College of Marine Science and Technology, Zhejiang Ocean University, Zhoushan 316022, China

*Correspondence: cuizg@ysfri.ac.cn

Supporting Figures:

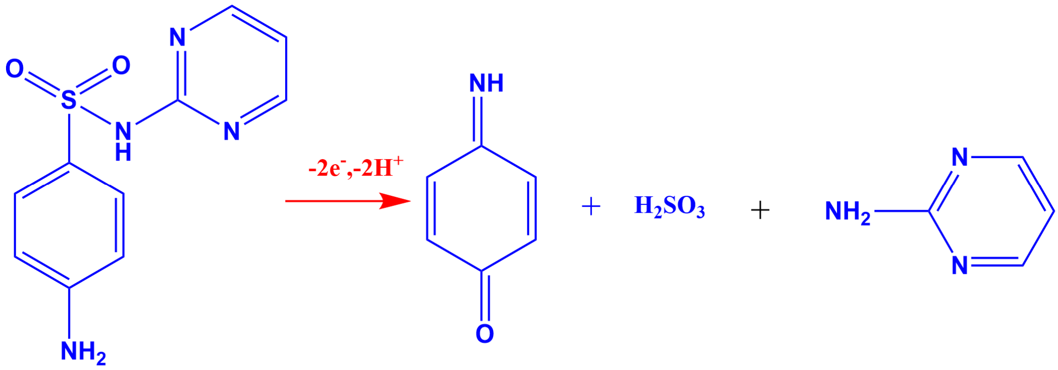

Figure. S1. Electrochemical oxidation mechanism of sulfadiazine.

Tables

Table S1. Analytical data comparison between SDZ detection and previous electrodes.

**Electrode**	**Electrolyte solution**	**LOD**	**Linear range**	**Ref.**
GCE	0.04 M B-R buffer (pH 6.8)	10.9 µM	62.7-340 µM	[1]
Carboxyl-MWCNTs/GCE	0.04 M B-R buffer (pH 2)	0.07µM	0.50-110 µM	[2]
MIP/GO@COF/GCE	0.2 M PBS (pH 7)	0.16 µM	0.5–200 µM	[3]
MWCNT-GCE	0.04 M B-R buffer (pH 7)	7.1 µM	10-2000 µM	[4]
GCE	0.1M ABS (pH 4)	6.14 µM	20-300 µM	This work

(GCE, glassy carbon electrode; MWCNT, multiwalled carbon nanotube; MIP, molecularly imprinted polymer; GO@COF, grapheneoxide@covalentorganic framework; B-R buffer, Britton–Robinson buffer; PBS, phosphate buffer; ABS, acetic acid-sodium acetate buffer solution).

Supporting references

1. Braga, O. C.; Campestrini, I.; Vieira, I. C.; Spinelli, A., Sulfadiazine determination in pharmaceuticals by electrochemical reduction on a glassy carbon electrode. *Journal of the Brazilian Chemical Society* **2010,** 21, 813-820.
2. He, B.-S.; Chen, W.-B., Voltammetric determination of sulfonamides with a modified glassy carbon electrode using carboxyl multiwalled carbon nanotubes. *Journal of the Brazilian Chemical Society* **2016,** 27, 2216-2225.
3. Sun, Y.; He, J.; Waterhouse, G. I. N.; Xu, L.; Zhang, H.; Qiao, X.; Xu, Z., A selective molecularly imprinted electrochemical sensor with GO@COF signal amplification for the simultaneous determination of sulfadiazine and acetaminophen. *Sensors and Actuators B: Chemical* **2019,** 300, 126993.
4. Fotouhi, L.; Hashkavayi, A. B.; Heravi, M. M., Electrochemical behaviour and voltammetric determination of sulphadiazine using a multi-walled carbon nanotube composite film-glassy carbon electrode. *Journal of Experimental Nanoscience* **2013,** 8, (7-8), 947-956.
